# Energetic and health effects of protein overconsumption constrain dietary adaptation in an apex predator

**DOI:** 10.1038/s41598-021-94917-8

**Published:** 2021-07-28

**Authors:** Karyn D. Rode, Charles T. Robbins, Craig A. Stricker, Brian D. Taras, Troy N. Tollefson

**Affiliations:** 1 U.S. Geological Survey, Alaska Science Center, 4210 University Drive, Anchorage, AK 99508 USA; 2 School of the Environment and School of Biological Sciences, Washington State University, Pullman, WA 99164 USA; 3 U.S. Geological Survey, Fort Collins Science Center, Fort Collins, CO 80526 USA; 4 Division of Wildlife Conservation, Alaska Department of Fish and Game, Fairbanks, AK 99701 USA; 5 Mazuri Exotic Animal Nutrition, Land O’Lakes, Inc., St. Louis, MO 63166 USA

**Keywords:** Conservation biology, Ecophysiology, Evolutionary ecology, Diseases, Kidney diseases, Liver diseases

## Abstract

Studies of predator feeding ecology commonly focus on energy intake. However, captive predators have been documented to selectively feed to optimize macronutrient intake. As many apex predators experience environmental changes that affect prey availability, limitations on selective feeding can affect energetics and health. We estimated the protein:fat ratio of diets consumed by wild polar bears using a novel isotope-based approach, measured protein:fat ratios selected by zoo polar bears offered dietary choice and examined potential energetic and health consequences of overconsuming protein. Dietary protein levels selected by wild and zoo polar bears were low and similar to selection observed in omnivorous brown bears, which reduced energy intake requirements by 70% compared with lean meat diets. Higher-protein diets fed to zoo polar bears during normal care were concurrent with high rates of mortality from kidney disease and liver cancer. Our results suggest that polar bears have low protein requirements and that limitations on selective consumption of marine mammal blubber consequent to climate change could meaningfully increase their energetic costs. Although bear protein requirements appear lower than those of other carnivores, the energetic and health consequences of protein overconsumption identified in this study have the potential to affect a wide range of taxa.

## Introduction

Understanding the physiological limitations of a species is critical to determining potential adaptation to environmental change^1^. Although responses to environmental change are often predicted based on currently observed relationships between a species and its environment and the existing range of behaviors, the ability to respond to environmental change is ultimately a function of physiological constraints that may or may not be apparent under current conditions^1,2^. Nutrition is an important component of physiology that, like other physiological limitations associated with metabolism, affects wildlife behavior and ultimately population dynamics via reproduction and survival^3,4^.

Evolutionary history plays an important role in determining constraints on nutritional ecology^5^. For example, felids which diverged from other species within the order Carnivora differ in requirements for amino acids, fatty acids, and vitamins as a result of their evolution to consume a high-protein, meat-only diet^5^. Similarly, the ability to extract nutrients from fibrous plants depends on the evolution of gut morphology and microflora that improve digestive efficiency^6^. When food resources available to a species are altered, their ability to withstand environmental change is a function of the degree to which altered diets can continue to meet energetic and nutritional needs^7^. Understanding a species’ nutritional physiology in light of their evolutionary history has important implications both for free-ranging populations and those in managed care^8^.

Ursids are among the least specialized carnivores. The family includes herbivorous giant pandas (*Ailuropoda melanoleuca*), six omnivorous species [e.g., brown bears (*Ursus arctos*), American black bears (*U. americanus*), sun bears (*Helarctos malayanus*)], and the carnivorous polar bear (*U. maritimus*). Their more generalized diets allow them to utilize diverse food resources from tropical forests to the Arctic sea ice. Recent studies indicate that despite diverse diets, bears optimize protein levels to simultaneously meet minimal requirements while avoiding the increased energetic costs associated with metabolizing protein consumed at levels above physiological requirements into urea to excrete excess nitrogen^9–11^. For example, coastal Alaskan brown bears congregate at streams where they have access to easily caught and abundant spawning salmon, yet, bears leave streams to spend 6–10 h/day, or ~ 50–90% of their foraging effort, eating small, dispersed, carbohydrate-rich berries^10^. By consuming both salmon and berries, brown bears optimize the protein:energy ratio such that it increases the rate of body mass gained by 72% compared to salmon-only diets^10^. Giant pandas are strict herbivores that increase dietary protein content by selectively consuming plant parts, but it is unclear whether dietary protein levels are similar to or exceed the lower levels consumed by other ursids^12,13^. Polar bears are strictly carnivorous on the sea ice, but they appear to preferentially consume the blubber of lipid-rich marine mammal prey which would reduce their protein intake below that of typical strict carnivores while maximizing energy intake^14^. Thus, among ursids maintaining lower levels of protein intake appears to play an important role in diet selection and nutrition.

Polar bears occupy a unique niche in the animal kingdom as the only species in the world that inhabits the Arctic sea ice and extracts their nutrition from marine mammals that live beneath the ice surface. Although they are the most carnivorous ursid, they evolved from omnivorous brown bears 150,000–600,000 years ago^15^. Studies of polar bear feeding ecology have focused on whether or not a bear has fed recently or the prey composition of the diet, and rarely, on the dietary nutritional composition. This narrow view of feeding ecology is common in studies of marine apex predators and other carnivores^16,17^. The assumption that carnivores “forage for prey” rather than in response to more specific nutritional requirements has recently been questioned^16,18^.

Global-warming induced sea ice loss in the Arctic is changing polar bear access to prey and prey composition^19,20^. Even during seasons when sea ice is not limiting, polar bears in some areas have experienced substantial declines in access to prey. For example, in the northern and southern Beaufort Sea subpopulations, the proportion of adult females that had not fed while on the sea ice in the past 7–10 days in March and April increased from 13 to 33% and 30 to 42%, respectively for the two subpopulations, between 1983 and 2016^20^. Declines in predation success reduce the opportunity to preferentially consume high fat blubber. Similarly, polar bears are increasingly using terrestrial habitats where available foods are carbohydrate-based and higher in protein, such as plants, lean meat, and bird eggs^21^.

An important factor that will affect the ability of polar bears to adapt to reduced availability of their marine mammal prey is whether they have retained the need to consume a relatively low protein diet similar to other ursids or evolved sufficiently towards strict carnivory that increased consumption of protein will not increase their energetic needs or affect long term health. We hypothesized that polar bears have retained the physiological niche of omnivorous brown bears and therefore consume lower protein diets to avoid the increased energetic costs associated with excretion of nitrogen when protein is consumed above physiological requirements^9–11^. If true, increased consumption of high protein diets would have important consequences for polar bear energetics and further support the implications of upper limits on protein consumption in carnivores. To address this hypothesis, we (a) estimated the dietary protein and fat content of diets consumed by wild polar bears in the Chukchi Sea subpopulation where prey is not currently limiting^21,22^, (b) measured the protein to fat dietary content selected by zoo polar bears that have never had access to high-fat marine prey to determine if protein:fat selection is genetically and physiologically based, and (c) evaluated the potential health consequences (e.g., energetic costs, diseases and lifespan) of consuming high protein diets that might affect wild polar bears when prey availability is reduced and be important in diet formulation for polar bears in managed care.

## Results

### Macronutrient: proportions consumed by wild and zoo polar bears

Polar bears in the Chukchi Sea subpopulation, where prey is not currently limiting, consumed diets consisting of 66–74% marine mammal blubber with little variation across sex and age classes (n = 229 adult and subadult bears) (Table 1). Fat content of blubber ranged from 85.4 to 95.0% across prey species (n = 18), whereas the fat content of muscle ranged from 7.9 to 9.3%^23^. The fat proportions in the diets of male and female polar bears in the Chukchi Sea from 2008 to 2017 averaged 67 ± 4% (± stdev across age classes of males) and 70 ± 1% (female) of dry matter, respectively (Table 2). Because fats are more energy dense than proteins, the metabolizable energy contents of male diets were 81 ± 3% fat and 19 ± 3% protein and female diets 83 ± 1% fat and 17 ± 0% protein (Table 2).

**Table 1:** Mean percentage (standard deviation) contributions of prey muscle and blubber to the diets of male and female polar bears in the Chukchi Sea based on a bulk isotope model using carbon and nitrogen isotopes in polar bear hair and prey muscle and blubber tissues. Dietary percentages for prey tissues that could not be differentiated in the model due to overlap in their isotopic signatures were combined. Sample sizes (n) for each sex and age class are provided in the second row. Adult bears are age > 10 years, young adults are age 5–10, and subadults are independent bears age 2–4.

Prey species	Adult females	Adult males	Young adult females	Young adult males	Subadult females	Subadult males	Fat content
N	53	52	34	47	16	27	
Bearded seal blubber	26 (15)	21 (11)	27 (16)	24 (13)	22 (16)	25 (17)	93.8
Ringed seal and Beluga whale blubber	23 (11)	22 (8)	21 (11	22 (10)	32 (14)	20 (11)	95.0
Ringed seal and Bearded seal pup muscle	23 (5)	30 (5)	22 (5)	24 (5)	25 (6)	20 (6)	8.0^a^
Walrus non-calf blubber	9 (9)	8 (7)	11 (12)	9 (9)	7 (8)	12 (14)	93.5
Bearded seal, Ringed seal, and Beluga muscle	5 (3)	5 (3)	5 (3)	6 (3)	3 (2)	6 (4)	8.3^a^
Walrus calf blubber	5 (6)	6 (5)	5 (7)	6 (6)	4 (5)	8 (10)	79.0
Bowhead whale blubber	6 (7)	5 (4)	5 (6)	5 (6)	6 (1)	5 (6)	93.4
Gray whale blubber	4 (4)	4 (3)	4 (5)	4 (5)	2 (3)	5 (7)	85.4
Total blubber	73 (6)	66 (5)	73 (7)	70 (6)	73 (6)	74 (7)	

^a^Data from Yurkowski et al.^23^. For combined prey muscle we assumed seal contributed more than beluga whale and therefore used a weighted value from the range of 7.9% for ringed seals and 9.3% for beluga whale.

**Table 2:** Fat and protein intake of 9 zoo polar bears and 5 captive brown bears fed ad libitum options of meat and fat (lard or salmon oil) and free-ranging polar bears in the Chukchi (n = 229) consuming diets of marine mammal prey. Zoo polar bears were provided lean meat and lard. Brown bears were provided salmon and salmon oil^11^. Standard deviations were calculated for the dry matter intake of fat and protein of Chukchi Sea polar bears using standard deviations of individual dietary components in Table 1 with respective proportional protein and fat content of prey tissues.

Zoo	Age	Sex	% of dry matter intake		% of metabolizable energy	
			Fat	Protein	Fat	Protein
Alaska Zoo, Anchorage, AK	16	F	72	28	85	15
Brookfield Zoo, Chicago, IL	13	M	67	33	81	19
Columbus Zoo, Columbus, OH	13	F	54	46	71	29
	20	M	59	41	75	25
Como Park Zoo and Conservatory, St. Paul, MN	24	M	58	42	75	25
	24	M	63	37	78	22
Detroit Zoo, Detroit, MI	14	M	71	29	84	16
	6	F	55	45	72	28
Seneca Park Zoo, Rochester, NY	24	F	50	50	61	39
Average (stdev) for Zoo bears			60 ± 9	40 ± 9	76 ± 7	24 ± 7
Brown bears (salmon/salmon oil)			66 ± 5	34 ± 5	80 ± 3	20 ± 3
Chukchi Sea adult polar bears		F	69 ± 8	31 ± 2	83	17
		M	63 ± 5	37 ± 2	78	21
Chukchi Sea young adult bears		F	70 ± 8	30 ± 2	82	17
		M	67 ± 8	33 ± 2	81	19
Chukchi Sea subadult bears		F	70 ± 8	30 ± 2	83	17
		M	71 ± 9	29 ± 2	84	16
Average for Chukchi Sea bears			68	32	82	18

Diets fed to polar bears in North American zoos (n = 10) during normal care contained 36 ± 16% fish on a fresh weight basis, 33 ± 15% raw meat and commercially prepared meat and fat mixtures, 12 ± 12% extruded dry pellets, 16 ± 21% fruits and vegetables, and 6 ± 6% oils and fats. Because the diets are predominantly low-fat fish and meat products, they average 37 ± 12% fat (range 22–67%), 51 ± 8% protein (range 29–62%), and 12 ± 5% digestible carbohydrate (range 4–25%) on a dry matter basis. This corresponded to diets of 55 ± 12% fat (range 36–82%), 37 ± 8% protein (range 16–47%), and 8 ± 5% carbohydrate (range 2–20%) on a metabolizable energy basis.

Zoo polar bears given ad libitum access to fat and protein selected diets containing 60 ± 9% fat and 40 ± 9% protein on a dry matter basis and 76 ± 7% fat and 24 ± 7% protein on a metabolizable energy basis (Table 2). The proportion of fat selected by zoo bears was 8% less as a percent of dry matter (F_1,14_ = 4.9, p = 0.04) and 7% less as a percent of metabolizable energy (F_1,14_ = 5.8, p = 0.03) than that consumed by wild polar bears. Percent of fat consumed did not exhibit a trend during the 14-day trials (t = − 1.8, p = 0.09). Male and female polar bears used in the study averaged 481 ± 29 kg (n = 5) and 301 ± 53 kg (n = 4), respectively, all of which were adults.

**Figure 1:**
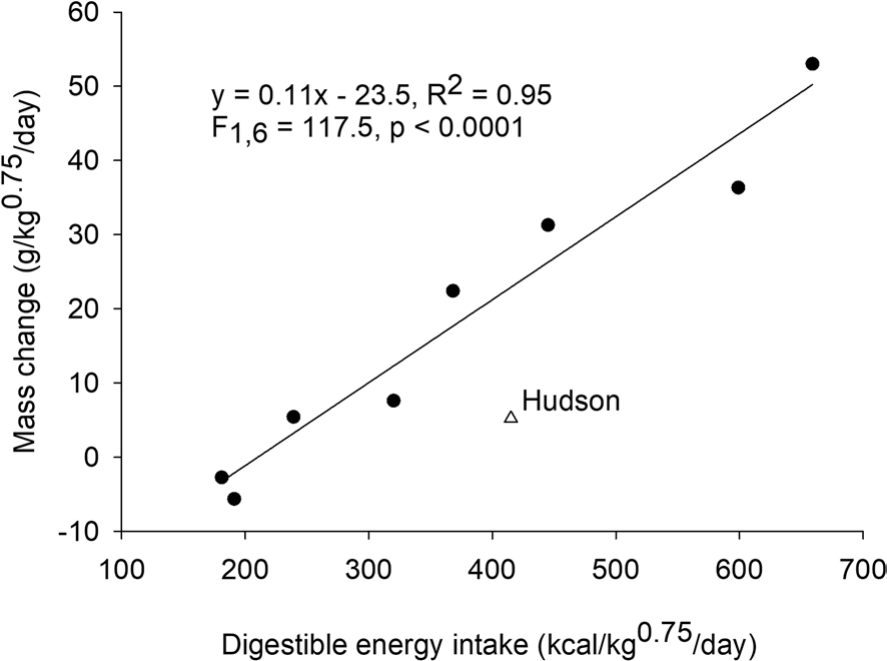
Relationship between digestible energy intake and mass change of 9 polar bears fed ad libitum lean meat and lard. Energy intake and mass change is presented relative to body mass scaled to metabolic rate (i.e., kg^0.75^). An adult male (Hudson) at the Brookfield Zoo was reported to have paced extensively during the trial while isolated from an adult female and was not included in the regression.

**Figure 2:**
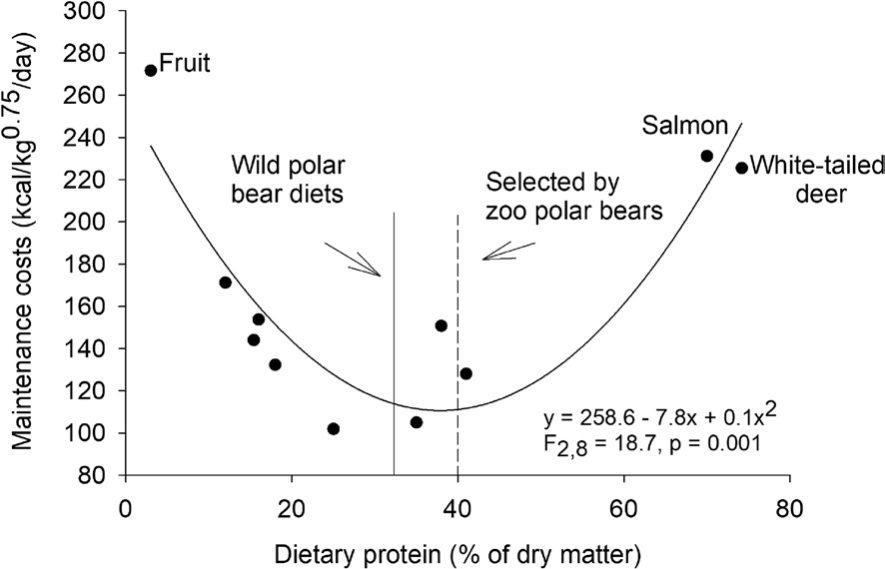
The non-linear relationship between dietary protein content and the energy required to maintain body mass (maintenance cost) for brown bears fed diets varying in dietary protein content (data points) in comparison to the dietary protein levels selected and consumed by wild and zoo polar bears. Low and high protein diets are associated with high maintenance energy costs. The dietary protein content selected by wild polar bears feeding on marine mammals as indicated by the solid vertical line, and the dietary protein content selected by zoo polar bears in feeding studies with ad libitum access to lean meat and lard as indicated by the dashed vertical line were within the range associated with minimization of maintenance energy costs. Brown bear diets ranged from 100% fruit (3% protein) to 100% salmon (61% protein) or 100% white-tailed deer^9–11,24^. Reference^24^ did not report meat protein content, so the protein content of white-tailed deer meat was determined from Ref.^25^. Digestible dry matter was converted to kcal using dry matter digestibility of deer for brown bears from Ref.^26^.

Relating the mass gain of the zoo polar bears during ad libitum selection trials to digestible energy intake (Fig. 1) allowed comparison of the metabolic efficiency of the diet selected by zoo polar bears with efficiency of mass gain in brown bears fed a range of protein levels in previous studies. Although the digestible energy intake of the zoo polar bears at 0 mass gain (214 kcal/kg0.75/day) was approximately double that of brown bears (Fig. 2), their efficiency of mass gain (0.11 g/kg digestible energy intake) was identical to that for brown bears at the same dietary protein content (relationship between brown bear dietary protein content as percent of dry matter (x) and efficiency of mass gain (y): y = 0.1370–0.0007x, R^2^ = 0.52, F_1,8_ = 8.7, p = 0.02). The higher energetic cost for maintaining body mass (maintenance energy cost determined by solving the equation of the line in Fig. 1 for y = 0) of zoo polar bears than brown bears was expected due to the larger exhibits available for zoo polar bears to roam compared to the habitat for the captive brown bears in previous studies. Further, while efforts could be made to standardize energetic costs of brown bears in previous studies, such controls were not possible with zoo polar bears on exhibit. Thus, both wild and zoo polar bears selected protein and fat at levels consistent with minimizing maintenance energy costs and higher efficiencies of mass gain as previously observed in brown bears (Fig. 2).

### Health indicators, lifespan, and causes of death in wild and zoo polar bears

The average age of adult male and female polar bears (> 4 years of age) captured in two subpopulations that range in Alaska (the southern Beaufort and Chukchi Sea subpopulations) from 1983 to 2017 was 11.5 ± 5.2 (n = 982) and 11.3 ± 5.1 years (n = 640), respectively. The oldest females captured in the two subpopulations were 29 in the southern Beaufort Sea and 30 in the Chukchi Sea and the oldest males were 28 in the southern Beaufort Sea and 27 in the Chukchi Sea, respectively. Only 6.5% of 1622 adult bears captured in the two subpopulations between 1983 and 2017 were over the age of 20.

During the 6 years from 2015 to 2020, 19 adult polar bears died in American zoos at an average age of 27 ± 6 years. Kidney disease was listed as a cause of death for 37% (7/19) of zoo polar bears and liver disease or cancer was listed as a cause of death for 32% (6/19) (Supplementary Table S1). Liver cancer and kidney disease co-occurred in 1 bear. Bears that died solely of kidney disease or kidney disease co-occurring with heart disease (but not liver disease and cancer) were, on average, 10 years younger (22 ± 5 years; n = 5) than bears that died of other causes (32 ± 5 years; n = 8; one-way ANOVA: F_2,14_ = 7.7, p = 0.006; Bonferroni post-hoc tests: p = 0.005). Bears listed with the cause of death as liver disease or cancer were 28 ± 4 years old (n = 5) which did not differ from the age of bears dying of other causes (post-hoc test: p = 0.36).

Serum urea (15.7 ± 12.2 mg/dl; range = 1–85.5; n = 628) and creatinine (1.1 ± 0.4 mg/dl; range = 0.3–3.5; n = 628) levels of wild adult polar bears sampled in the southern Beaufort and Chukchi Sea subpopulations were well below values observed in zoo polar bears with chronic kidney disease (serum urea: 180.1 ± 77.8 mg/dl; n = 9; creatinine: 14.8 ± 12.7 mg/dl; n = 90)^27^. Mean phosphorus levels of wild adult bears (5.6 ± 1.3 mg/dl; n = 628) were also below those of polar bears with kidney disease (14.5 ± 6.9 mg/dl; n = 8)^27^, but 3 wild bears exhibited phosphorus levels above 10.0 (range 1.7–13.2 mg/dl). For wild polar bears age 20 and older (n = 32), urea averaged 13.8 ± 11.8 (range 3–40), creatinine averaged 1.1 ± 0.3 (0.7–2.0), and phosphorus averaged 5.7 ± 1.9 (range 2.9–13.2). Urea, creatinine, and phosphorus levels did not differ between bears < 20 years old and bears age 20 and older (p > 0.05).

## Discussion

Wild and zoo polar bears and captive brown bears strongly preferred diets high in fat and low in protein (Ref.^11^; this study), suggesting that the macronutrient requirements of polar bears closely align with those of the omnivorous brown bear. Because the largest males do most of the breeding and the fattest females have the highest probability of producing surviving cubs^28^, evolution should have favored bears that select the most efficient balance of macronutrients. The efficiency of mass gain on high fat diets may have similarly played a role in the evolution of polar bear milk consumed by cubs between 4 and 16 months of age which has an average dry matter content of 23.6% protein, 69.7% fat, and 3.1% carbohydrates, or a metabolizable energy ratio of 14% protein: 82% fat: 4% carbohydrate^29^. Although other marine mammals have high fat milks, most lactate for much shorter periods (< 2 months) than polar bears to promote rapid growth of young required for swimming and at-sea feeding. Consistency in macronutrient composition of milk for young, rapidly growing cubs and diets selected by fully grown adults suggests that these protein levels meet requirements of both growing and fully grown individuals of both sexes. High fat milk and selective feeding on blubber by polar bears after weaning also maximize energy intake supporting polar bears to be the largest of the ursid species.

Although few studies of the feeding ecology of predators consider the implications of macronutrient consumption, consuming protein in excess of requirements increases energy intake requirements in a variety of taxa because of the need to metabolize and excrete excess nitrogen (e.g., mink *Neovison vison*^30^; Stellar sea lions^31^; harp seals *Phoca groenlandica*^32^; harbor seals *Phoca vituline*^33^; northern fur seals *Callorhinus ursinus*^34^; pigs^35^; humans^36^). Selected protein levels vary across carnivores in which data are available, but some patterns are consistent with phylogeny. Among species in the Order Carnivora, cats (i.e., felids) and hyenas diverged from all other carnivores 35–38 million years ago^37^ and specialize in the consumption of high protein, lean meat diets (e.g., ≥ 50% of metabolizable energy (ME) as protein^18,38^) whereas other carnivores select diets lower in protein (< 40% of ME as protein) and exhibit higher nitrogen excretion and metabolic costs when consuming diets above selected levels (canids^39^; harbor seals^33^; northern fur seals^34^; mink^30^; brown bears^11^). For example, the percent of metabolizable energy available to northern fur seals declined from 83 to 71% on diets containing 36% and 63% of energy from protein, respectively^34^. Similarly, declines in Steller sea lions (*Eumetopias jubatus*) in Alaska have been linked to the increased metabolic cost of consuming and metabolizing higher-protein, lower fat prey (54% of ME as protein; pollock; *Theragra chalcogramma*) consequent to reduced availability of lower protein Pacific herring (36% of ME as protein; *Clupea pallasi*)^40^. The energetic consequences of consuming protein above physiological requirements likely also explains observed selective feeding for fat-rich prey parts in non-felid carnivores (e.g., harbor seals^41^).

In addition to affecting energetic requirements, overconsumption of protein has been associated with negative effects on kidney and liver health. Pigs and rats fed diets with 35% of energy from protein developed kidney damage and exhibited higher kidney weights and higher creatinine clearance^42,43^ as the kidneys worked to excrete excess nitrogen. In humans, diets high in animal protein impair kidney function, increase the propensity for kidney disease, and increase the progression of kidney disease^44^. Protein intake above requirements even in cats has been identified as a precursor to chronic kidney disease^45^. Similarly, high protein intake has been associated with increased risk of liver cancer in humans^46^. Thus, excretion of nitrogen in excess of requirements is associated with effects on kidney and liver health across a variety of taxa.

Current zoo polar bear diets are higher in protein (51% dry matter) than those consumed by wild polar bears (32%) and selected by zoo polar bears (40%). Because zoo polar bears are also fed carbohydrates which have lower calorie density than fats, protein contributes approximately twice as much to their caloric intake (37%) when compared to diets consumed by wild bears (18%) and selected by zoo bears in ad libitum trials (24%). Chronic kidney disease (29% of zoo polar bear mortalities from 1995 to 2011^27^; 37% this study) and liver disease and cancers (32%) are the primary causes of death in zoo polar bears, the former of which may have increased in prevalence in recent years (this study). Zoo polar bears dying of kidney disease died 10 years earlier than bears dying of other causes and 5–9 years earlier than the maximum age achieved by wild polar bears. Serum urea, creatinine, and phosphorus levels measured in over 600 wild adult polar bears (this study) and among 35 wild polar bears in Svalbard^47^ were consistently well below levels documented in zoo polar bears with chronic kidney disease^27^, suggesting that kidney disease is not an affliction in wild polar bears. Thus, the potential that high protein intake may affect metabolism and kidney and liver health suggests the need for careful consideration of protein levels in formulated diets for carnivores (i.e., species in the order carnivora)—particularly ursids which have a long evolutionary history of omnivory and diets dominated by carbohydrates or fat.

Achieving optimal protein intake has the potential to become an increasing challenge for wild polar bears. The most common prey of polar bears is ringed seals (*Pusa hispida*), which contribute 60–80% of the diet but vary widely in energy content with changes in blubber composition^21,48^. Adult ringed seals can range from 29 to 50% blubber^49^, and ringed seals of all ages range in total fat content of 34–76% of dry matter and 52–85% of total body energy^14^. Thus, if polar bears consume entire ringed seals as opposed to selectively feeding on blubber, dietary protein can be up to 66% of dry matter intake and within ranges associated with increased maintenance energy requirements (Fig. 2). This could be occurring in areas where access to seals appears to be reduced, including the southern Beaufort Sea^20^ and Hudson Bay^50^. Furthermore, in some areas polar bears are spending longer periods on land where they scavenge on the remains of subsistence harvested bowhead whales in which the blubber has been removed^51^. As polar bears increasingly use terrestrial habitats throughout the Arctic^52^, they will be challenged to simultaneously meet their high total energy requirements and avoid the increased energetic costs associated with metabolizing protein-rich terrestrial mammals and bird eggs and the reduced energy intake from carbohydrate-dominated plants as opposed to lipid-rich marine mammals^53^. Thus, their evolutionary divergence from brown bears and the associated physiological constraints on protein intake are likely an additional factor limiting the adaptation of polar bears to sea ice loss.

The energetic and health consequences of consuming protein in excess of physiological requirements appear to be near-universal across taxa consequent to similar physiological processes for coping with excess nitrogen that require energy and tax the liver and kidneys in ways that can lead to disease. In animal nutrition, protein is primarily considered per its role as a limiting resource, as is the case for herbivores. When protein is limited it can affect species behavior, health, and population dynamics^54^. Yet, our results, along with those of others^34,40,55^, suggest that the implications of protein overconsumption may play an equally important role in affecting behavior, health, and conservation.

## Methods

### Estimation of dietary fat consumption of wild polar bears

We used stable isotopes of carbon and nitrogen in polar bear hair and prey bulk (i.e., not lipid-extracted) blubber and muscle samples to estimate the contribution of prey blubber to the diets of polar bears in the Chukchi Sea. The Chukchi Sea subpopulation ranges on sea ice over productive shallow continental shelf waters between the northwest coast of Alaska and the northeast coast of Russia. Recent studies indicate that this population is not currently prey-limited and has maintained body condition and survival rates that support a stable population^20–22,56^. Similarly, populations of the two primary prey species, bearded (*Erignathus barbatus*) and ringed seals, appear to be stable^57^.

Blubber has a substantially lower δ^13^C and a marginally higher δ^15^N content than muscle^58^ resulting in these tissues being isotopically distinct among polar bear prey. For example, bulk muscle and blubber of ringed seal sampled in the Alaskan Chukchi Sea had an average δ^13^C of − 18.9 ± 0.9 (stdev; n = 43) and − 25.4 ± 0.7 (n = 6), respectively (Supplementary Table S2). Other prey species similarly exhibited a substantial difference in δ^13^C between muscle and blubber that, in most cases, exceeds differences between prey species (Supplementary Table S2). We capitalized on these isotopic differences to estimate contributions of prey blubber and muscle tissue to polar bear diets inferred from hair.

Guard hair was collected from the upper forearm of polar bears captured on sea ice in the spring (i.e. March–May) in the Alaskan portion of the Chukchi Sea from 2008 to 2017 and analyzed for δ^13^C and δ^15^N. Bears were located by helicopter and immobilized with zolazepam-tiletamine (Telazol) administered via a dart. Immobilization and sampling was approved and carried out in accordance with the U.S. Marine Mammal Protection and Endangered Species Acts, under the U.S. Fish and Wildlife Service (USFWS) permit number MA 046081 and animal handling protocols established by the USFWS Region 7 Institutional Animal Care and Use Committee.

Hairs collected from polar bears in the spring primarily represent the period of maximum hair growth that occurred the prior June–August with some additional growth occurring in September and October^59^. A prey carbon and nitrogen isotope library was generated for muscle and blubber of known prey species collected from subsistence harvested marine mammals and carcasses of polar bear hunted prey encountered during annual capture efforts (Supplementary Table S2). These included ringed seal, bearded seal, walrus (*Odobenus rosmarus*), beluga whale (*Delphinapterus leucas*), bowhead whale (*Balaena mysticetus*), and gray whale (*Eschrichtius robustus*)^21,60^. Pups and calves of bearded seal, ringed seal, and walrus were also included as separate prey items due to the unique isotopic signature of their muscle in comparisons to non-nursing, older individuals of each species^61^. Details on polar hair and prey muscle and blubber preparation and isotopic analyses are provided in Supplementary Appendix S1.

The proportion of blubber and muscle consumed from each food item was estimated using the Bayesian mixing model framework, MixSIAR, implemented in R^62^. We used bulk trophic enrichment factors (TEFs) derived for hair from zoo polar bears fed diets that mimicked the carnivorous (i.e. protein and fat only), marine diets consumed by free-ranging polar bears^63^. The TEFs were corrected for differential C concentration in dietary fat and protein assuming these macronutrients were composed of 74% and 42% C, respectively^63^. This resulted in bulk diet TEFs of Δ^13^C = 7.72 ± 0.60‰ and Δ^15^N = 1.47 ± 1.07‰. We included the combination of sex and age class as a categorical variable with six levels, including males and females of three age classes: subadults (independent bears age 2–4), young adults (age 5–10) and adults (age 11+). These classes differentiate reproductively mature individuals (age 5+) from non-reproductively mature (2–4) and full-grown males (11+) from males that are continuing to increase in length and body mass (age 5–10) as described by Ref.^21^. Dependent offspring were excluded from the analysis.

We initially included the muscle and blubber of all prey in diet models. However, the results indicated that there was a 95% chance that the muscle of walrus calves and non-calves, gray whale and bowhead whale each contributed ≤ 1% to the total diet. Because many of these are large prey species in which polar bears would be likely to select exclusively for blubber, and the initial results suggested low dietary proportions, these prey tissues were removed from the final diet model. Although these were all prey muscle, removing these species tissues from the diet estimates had minimal effect on our estimate of percent dietary fat (i.e., the model with all prey muscle components generated a fat estimate of 67% dietary fat as dry matter compared to 68% dietary fat when these components were excluded).

Not all tissues from prey species could be differentiated isotopically because both their δ^13^C and δ^15^N values overlapped. Therefore, we combined dietary proportions estimated from the model for tissues of species in which both δ^13^C and δ^15^N overlapped, including pup and non-pup blubber for bearded and ringed seals (i.e., the two species blubber differed but pups and non-pups of each species overlapped), muscle of ringed and bearded seal non-pups and beluga whale, and the muscle of ringed and bearded seal pups^62^. Thus, final prey proportions are combined among some species and tissues. The MixSIAR model generated average muscle and blubber prey proportions for each of the six combinations of polar bear sex and age class.

Dietary fat content was determined by summing the percent of fat contributed by muscle and blubber components of each species in the diet. Blubber fat contents were estimated per gravimetric analyses described in Supplementary Appendix S1 and supplemented with values provided by Ref.^23^. Because prey and hair samples are dried for isotope analysis, %blubber and %dietary fat estimates are on a dry matter basis. Standard deviations were calculated for the dry matter intake of fat and protein of Chukchi Sea polar bears using standard deviations of individual dietary components in Table 1 with respective proportional protein and fat content of prey tissues. Isotopic estimates from hair represent assimilated diet and therefore do not require correction for digestion and metabolism. The contribution of mean dietary fat and protein to metabolizable energy was determined using Atwater specific factors of 4.27 kcal/g dry matter for meat protein and 9.03 kcal/g dry matter for meat lipid^64^.

### Macronutrient composition of diets fed to polar bears at North American zoos

The protein, fat, and digestible carbohydrate (i.e., sugars and starch) content of diets fed to polar bears in 10 North American Zoos were estimated using nutrient analyses provided by the zoos, manufacturers’ data for the commercial diets, and nutrient composition tables such as that found at https://fdc.nal.usda.gov/ for foods commonly consumed by humans. When zoo diets varied seasonally, the average macronutrient content of the two diets was used to estimate the average across zoos. The metabolizable energy content of the diets fed to zoo polar bears was calculated by multiplying the total dry matter amount of protein, fat, and carbohydrate fed by Atwater specific factors of 4.1 kcal/g for proteins and carbohydrates and 9.0 kcal/g fat^11,64^ rather than the meat-specific values since the source of macronutrients from animal or plant matter varied.

### Feeding trials to measure macronutrient selection of zoo polar bears

**Figure 3:**
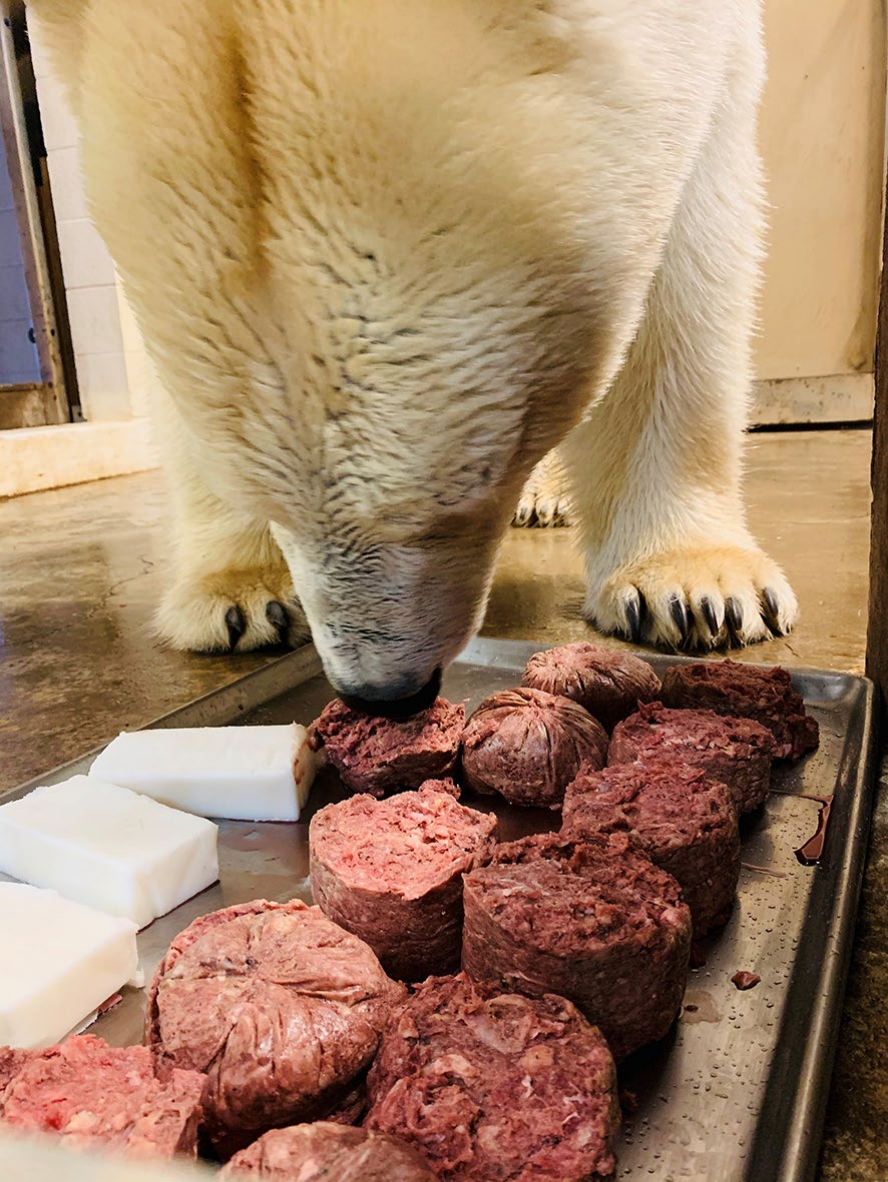
A female polar bear at the Columbus zoo selects among lean meat and lard food options (Photo c/o Devon Sabo).

Fourteen-day macronutrient selection trials were conducted with 9 polar bears (5 males and 4 females) in cooperation with 6 U.S. zoos. This research was approved and carried out in accordance with a letter of authorization from the USFWS issued October 18, 2018 and study protocols were approved by the U.S. Geological Survey Alaska Science Center’s Animal Care and Use Committee (IACUC) and the American Zoological Association’s Polar Bear Research Council. Bears were given the opportunity to consume either lean meat (e.g., lean beef, horse meat, or a low fat commercial canine diet such as Nebraska Premium Canine) or fat (e.g., lard or trimmed beef or pork fat) presented in separate piles ad libitum during 2–3 feedings per day (Fig. 3). A 4-day dietary adjustment period occurred prior to each 14-day trial during which each bear was given increasing amounts of both foods. By the 3rd or 4th day of the adjustment period, the amount offered was increased to ad libitum such that surplus food was still available at the end of each meal. Each bear was weighed prior to their first feeding of the 14-day trial and before feeding on the morning following the last day of the trial. The meat and fat offered and rejected were weighed each day to determine the total amount consumed. Samples of each food were collected throughout the study and stored frozen immediately after collection and during shipping to Washington State University for nutritional analyses. Samples were freeze-dried to determine dry matter content, ground and analyzed to estimate crude protein content (N X 6.25) via a nitrogen-carbon analyzer, fat content by ether extraction, and gross energy content by bomb calorimetry^11^. Carbohydrates were not measured because meat has < 1% soluble carbohydrates. Digestibility of fat and energy were estimated as 99% and 97%, respectively^65^. The metabolizable energy content of each bear’s selected diet was calculated by multiplying the total dry matter amount of protein and fat consumed during the 14-day study by the Atwater specific factors for meat protein and meat lipid used for wild bear diets.

We used a mixed effects model to test for changes in macronutrient preferences within and between bears during the 14-day trials. The dependent variable was % dietary fat, bear was a random effect, and trial day was a fixed effect for each bear (i.e., accounting for repeated measures for each bear). We used an ANOVA to test for differences between the macronutrient preferences of zoo polar bears and the macronutrient consumption of the six sex and age classes of wild polar bears. We used an α level of 0.05 to test for significance.

### Serum indicators of kidney function in wild and zoo polar bears

Blood samples were collected from 628 adult (age 5 + years) polar bears of both sexes captured in two polar bear subpopulations that range in Alaska (the Chukchi and southern Beaufort Sea subpopulations) from 2004 to 2017 and analyzed for serum urea, creatinine, and phosphorus levels to determine if levels were consistent with those of zoo polar bears exhibiting chronic kidney disease reported by Ref.^27^: serum urea (180.1 ± 77.8 mg/dl; n = 9), creatinine (14.8 ± 12.7 mg/dl; n = 9) and phosphorus levels (14.5 ± 6.9 mg/dl; n = 8). All wild bears were sampled between March and May. Blood was collected and stored unfrozen during transport to base camps where samples were centrifuged to remove serum. Serum was stored frozen until analysis. Both serum urea and creatinine have been found to be stable in blood samples for up to 10 years^20^. All samples in this study were analyzed within 10 years of collection. Thawed serum was analyzed for urea and creatinine levels using comprehensive rotors with an Abaxis vetscan (Abaxis, Inc., Union City, CA, USA^20^). Independent bears in both subpopulations were aged by counting the cementum annuli in a vestigial premolar. Dependent young were aged based on tooth eruption patterns which differ between first year cubs, yearlings, and two-year olds. Female polar bears typically wean cubs by the age of 2. We report averages, standard deviations, and ranges for all bears age 3 and older and bears age 20 and older.

### Lifespan and causes of death in wild and zoo polar bears

We examined the age of death and causes of mortality for polar bears held in zoos based on news reports. Mortality data for zoo polar bears were previously summarized for 1995–2011 by Ref.^27^. Thus, we report only deaths occurring after 2011. We compared the age at death among bears listed as dying of kidney disease or kidney disease co-occurring with heart disease (since heart disease is often a consequence of kidney disease; n = 5), bears listed as dying of liver cancer or disease (n = 5), and bears listed as dying of other causes (n = 8). We excluded two 19-year old bears that died of a bacterial infection and meningitis, both of which occurred at the same zoo, from the age comparison. Comparisons were made using a one-way ANOVA with a Bonferroni post-hoc test.

Natural mortalities of polar bears in the wild are rarely observed. Thus, direct information on the age and causes of mortality cannot be ascertained. Rather, we summarized the age structure of polar bears captured from the southern Beaufort Sea subpopulation from 1983 to 2017 and from the Chukchi Sea subpopulation from 2008 to 2017 to identify the proportion of bears in the subpopulation that reach various ages, as well as the maximum age achieved by wild bears in these two subpopulations. Both subpopulations are harvested by hunters from indigenous coastal communities. Harvest rates are estimated to be low (< 4% of the subpopulation) but are typically male-biased which may affect age distribution in older classes. Ages of captured bears was determined as described above.

Methods for all aspects of data collection from wild and zoo polar bears were carried out in accordance with relevant guidelines and regulations and approved as specified in the above sections relating to sampling of wild and zoo polar bears. In addition, all aspects of the study are reported here in accordance with ARRIVE guidelines (https://arriveguidelines.org).

## Supplementary Information

Supplementary Information.

## Data Availability

Data used in this study will be released simultaneous to publication at the following locations: Rode, K.D. 2021. Carbon and Nitrogen Isotope Concentrations in Polar Bear Hair and Prey from the Alaska Beaufort and Chukchi Seas, 1978–2019: U.S. Geological Survey data release, https://doi.org/10.5066/P9KM5FT2. USGS Alaska Science Center, Polar Bear Research Program. 2021, Protein and fat consumption of zoo polar bears in 14-day ad libitum trials 2019–2020: U.S. Geological Survey data release, https://doi.org/10.5066/P9W7MP0T.
