# Supplementary material for: Energetic and health effects of protein overconsumption constrain dietary adaptation in an apex predator
Source: Sci Rep. 2021 Jul 28;11:15309. doi: 10.1038/s41598-021-94917-8 (PMC8319126; doi:10.1038/s41598-021-94917-8)
Supplement: Supplementary file 1 — Supplementary Information. [file 41598_2021_94917_MOESM1_ESM.pdf]

Energetic and health effects of protein overconsumption constrain dietary adaptation in an apex predator

Karyn D. Rode, Charles T. Robbins, Craig A. Stricker, Brian D. Taras, Troy N. Tollefson

Supplementary Information

Supplementary Information

Table S1. Age, sex, and causes of death of zoo polar bears held in United States zoos since the earlier compilation of LaDouceur et al. (2014)

Age (years)	Sex	Date of Death	Cause of Death	Reference
34	F	October 2015	Urinary tract infection; mobility issues	Babay 2015
26	M	April 2015	Kidney disease, renal failure	McCauley 2015
31	M	July 2016	Liver cancer	Williams 2016a
33	F	November 2016	Ovarian cancer, early kidney and liver failure	Williams 2016b
29	F	September 2016	Liver and adrenal cancer, kidney and heart disease	Bethencourt 2016
26	M	January 2016	Lymphoma	Menchetti 2016
16	F	October 2017	Kidney disease, renal failure	Anon 2017
19	F	December 2017	Meningitis	Kelly 2018
26	M	December 2017	Kidney disease, renal failure	Blau 2017
19	F	April 2017	Kidney disease, renal failure	Koch 2017
29	M	April 2017	Liver cancer	Neese 2017
36	F	April 2017	Congestive heart failure	Dudnick 2017
21	F	April 2017	Chronic kidney and heart disease	H. Bissell, Sea World Parks & Entertainment, written communication; 1/28/2020
37	F	February 2018	Age	Madei 2018
28	F	March 2018	Liver and gallbladder disease	Freile 2018
21	F	February 2019	Liver cancer	Rizzo 2019

19	M	October 2019	Endocarditis, bacterial infection	Hollander 2019
24	M	August 2020	Neurological issues	Anon 2020
34	M	Sept 2020	Arthritis, gastrointestinal problems, dental and liver disease	Glenn 2020

References

Anon. 2017. Visitor favorite Kobe the polar bear euthanized at Lincoln Park Zoo. Chicago Sun-Times. October 20.

Anon. 2020. Buzz, Como Park Zoo polar bear diets at 24. August 1, St. Paul Star Tribune.

Babay, E. 2015. Philadelphia Zoo's Klondike the polar bear dies. Philadelphia Inquirer, October 23.

Bethencourt, D. 2016. Tundra, 29-year-old polar bear, dies at Detroit Zoo. Detroit Free Press, September 26.

Blau, R. 2017. Bronx Zoo's beloved Tundra—the last polar bear in NYC—dies at 26. New York Daily News, December 28.

Dudnick, L. 2017. SFZoo's only polar bear dies. San Francisco Examiner, April 14.

Freile, V.E. 2018. Seneca Park Zoo's only polar bear dies. Democrat and Chronicle, March 7.

Glenn, S. 2020. Oldest known polar bear in the world dies at Point Defiance Zoo. Tacoma news Tribune, September 1.

Hollander, Z. 2019. Lyutyik the polar bear, Alaska Zoo webcam star, has died. Anchorage Daily News, October 14.

Kelly, D. 2018. Ahpun the polar bear, a star attraction at the Alaska Zoo, has died. Anchorage Daily News, January 3.

Koch, M. 2017. Hogle Zoo's beloved 19-year-old polar bear Rizzo dies Sunday. Standard-Examiner, April 9.

LaDouceur, E. E. B., M. M. Garner, B. Davis, and F. Tseng. 2014. A retrospective study of end-stage renal disease in captive polar bears (*Ursus maritimus*). Journal of Zoo and Wildlife Medicine 45:69-77.

Lapin, T. 2017. Bronx Zoo's last polar bear euthanized due to health issues. New York Post, December 28.

Madei, P. 2018. Coldilocks, Philadelphia Zoo polar bear, euthanized after 'serious decline' in health. Philadelphia Inquirer, February 20.

McCauley, M.C. 2015. Magnet the polar bear dies from kidney disease at the Maryland Zoo. Baltimore Sun, April 28.

Menchetti, C. 2016. Zoo announces passing of male polar bear. Seneca Park Zoo. http://senecaparkzoo.org/wp-content/uploads/2016/03/Seneca-Parrk-Zoo-Announces-passing-of-male-polar-bear.pdf

Neese, A.W. 2017. Columbus zoo polar bear Nanuq dies. Columbus Dispatch, April 26.

Rizzo, T. 2019. Bam Bam, one of two polar bears at the Kansas City Zoo, has died. Kansas City Star, February 20.

Williams, K. 20161. Conrad, beloved Portland polar bear, dead at age 31. Oregonian, July 21.

Williams, K. 2016b. Oregon Zoo polar bear Tasul dead at age 31. Oregonian, November 18.

Table S2. Mean (± standard deviation) $\delta^{13}C$ and $\delta^{15}N$ concentrations in bulk (i.e., not lipid extracted) muscle and bulk blubber of polar bear prey species in the Chukchi Sea. $\delta^{13}C$ and $\delta^{15}N$ of bulk and lipid-extracted muscle samples did not differ. Thus, previously lipid-extracted muscle samples from collaborators were used to augment data where sample sizes were low for bulk analysis. Prey carbon and nitrogen isotope data are available in USGS (2021).

	**Muscle**			**Blubber**		
	**n**	**$\delta^{13}C$**	**$\delta^{15}N$**	**n**	**$\delta^{13}C$**	**$\delta^{15}N$**
**Ringed seal non-pup**	43	-18.9 (0.9)	17.0 (0.8)	6	-25.4 (0.7)	20.2 (0.9)
**Ringed seal pup**	5	-18.0 (1.2)	18.7 (0.9)	8	-25.9 (0.9)	20.1 (0.6)
**Bearded seal non-pup**	115	-17.8 (1.2)	16.5 (1.1)	4	-24.1 (0.6)	18.6 (1.5)
**Bearded seal pup**	7	-17.9 (0.6)	18.2 (0.7)	7	-24.1 (0.9)	18.8 (0.9)
**Walrus non-calf**	259	-17.0 (0.6)	12.6 (0.7)	7	-23.8 (08)	16.1 (0.7)
**Walrus calf**	1	-17.0	13.2	6	-22.5 (1.1)	17.5 (0.8)
**Beluga whale**	2	-18.8	17.6	3	-25.5 (0.2)	20.5 (0.2)
**Bowhead whale**	9	-21.4 (0.7)	12.4 (0.6)	6	-26.7 (0.3)	16.8 (0.8)
**Gray whale**	17	-17.3 (1.0)	12.0 (0.9)	1	-22.1	14.5

References

USGS Alaska Science Center, Polar Bear Research Program. 2021, Protein and fat consumption of zoo polar bears in 14-day ad libitum trials 2019-2020: U.S. Geological Survey data release, https://doi.org/10.5066/P9W7MP0T.

Appendix S1: Preparation and isotopic analysis of polar bear hair and prey blubber and muscle samples.

Hair was cleaned by soaking in three successive beakers containing 2:1 v/v chloroform:methanol (i.e. progressively cleaner with respect to organic contaminants) and air dried overnight. Dried hair was packaged into 4 x 6 mm silver capsules for mass spectrometry because polar bear guard hairs have a high tensile strength that precluded the typical use of tin capsules. Prey muscle and blubber samples were freeze-dried and homogenized. Blubber was sub-sampled and massed to fit inside of a 10 x 50 mm cellulose extraction thimble. Packed thimbles were extracted by Soxhlet distillation for five hours using a 2:1 chloroform:methanol solvent mixture. The fat residue plus solvent was retained, evaporated at room temperature under a light vacuum for approximately three days, and analyzed for weight % carbon and $\delta^{13}C$. The structural protein (which is primarily collagen) was retained, massed, and lyophilized. Prey blubber fat and structural protein, prey bulk muscle, and polar bear hair were analyzed for carbon and nitrogen elemental and isotopic compositions via conventional continuous flow isotope ratio mass spectrometry at several different laboratories. However, most measurements were conducted at the U.S. Geological Survey stable isotope laboratory (Denver, CO, USA; see Rode et al. 2016 for details on normalization and data quality and control). The relative proportions of fat and protein in blubber (i.e., gravimetric data) and their respective $\delta^{13}C$ and $\delta^{15}N$ were used to calculate 'bulk' blubber isotopic compositions.

Reference

Rode, K.D., Stricker, C.A., Erlenbach, J., Robbins, C.T., Cherry, S.G., Newsome, S.D. et al. Isotopic incorporation and the effects of fasting and dietary fat content on isotopic discrimination in large carnivorous mammals. Phys Biochem Zoology 89, 182-197 (2016). https://doi.org/10.1086/686490
